# 

**Published:** 1864-09

**Authors:** 


### CHICAGO MEDICAL JOURNAL.

**Terms, $2.00 per Annum.**

### CONTENTS OF THE SEPTEMBER NUMBER.

PAGE.

ORIGINAL COMMUNICATIONS.

Observations on "Disease." By J. A. A. .......... 383

"Bird's" Treatment of Prolapsus Uteri. By W. B. Slayter, M. D., of Chicago, Graduate Roy. Col. of Physicians of England, etc. .......... 389

Pessaries. By M. M. Eaton, M. D., of Peoria .......... 391

Clinical Lectures on Diseases of the Eye. By E. L. Holmes, M. D., of Chicago, Lecturer, on Diseases of the Eye and Ear in Rush Medical College—Chronic Conjunctivitis—The Lids .......... 393

SELECTED.

Clay as an Application to Ulcers .......... 400

A New Method of Treating Disease by controling the Circulation of the Blood in different parts of the body. By John Chapman, M. D., M. R. C. P .......... 401

Dislocation of the Hip Joint Successfully Reduced by Manipulation five months after the accident. By J. Newton Brown, M. D., San Jose. 409

Clinical Lecture on Diphtheria. By John T. Metcalfe, M. D., Professor Practice of Medicine University Medical College .......... 412

The case of Surgeon-General Hammond .......... 419

The Sanitary Commission .......... 421

The Origin of Cow-Pox and the Nature of Vaccine Virus .......... 425

Dissolving Power of the Pancreatic Juice .......... 426

Aggregate Lab r of Mankind .......... 426

Atropia: Its Action and Uses .......... 427

Grafting Animals .......... 427

